# Discovery of cell surface vimentin targeting mAb for direct disruption of GBM tumor initiating cells

**DOI:** 10.18632/oncotarget.12458

**Published:** 2016-10-04

**Authors:** Hyangsoon Noh, Jun Yan, Sungguan Hong, Ling-Yuan Kong, Konrad Gabrusiewicz, Xueqing Xia, Amy B. Heimberger, Shulin Li

**Affiliations:** ^1^ Department of Pediatrics–Research, The University of Texas MD Anderson Cancer Center, Houston, TX 77030, USA; ^2^ Department of Medicine, Baylor College of Medicine, Houston, TX 77030, USA; ^3^ Department of Neurosurgery, The University of Texas MD Anderson Cancer Center, Houston, TX 77030, USA

**Keywords:** cell surface vimentin, glioblastoma multiforme, tumor initiating cells, cancer therapeutic target

## Abstract

Intracellular vimentin overexpression has been associated with epithelial–mesenchymal transition, metastasis, invasion, and proliferation, but cell surface vimentin (CSV) is less understood. Furthermore, it remains unknown whether CSV can serve as a therapeutic target in CSV-expressing tumor cells. We found that CSV was present on glioblastoma multiforme (GBM) cancer stem cells and that CSV expression was associated with spheroid formation in those cells. A newly developed monoclonal antibody against CSV, 86C, specifically and significantly induced apoptosis and inhibited spheroid formation in GBM cells *in vitro*. The addition of 86C to GBM cells *in vitro* also led to rapid internalization of vimentin and decreased GBM cell viability. These findings were associated with an increase in caspase-3 activity, indicating activation of apoptosis. Finally, treatment with 86C inhibited GBM progression *in vivo*. In conclusion, CSV-expressing GBM cells have properties of tumor initiating cells, and targeting CSV with the monoclonal antibody 86C is a promising approach in the treatment of GBM.

## INTRODUCTION

Monoclonal antibody (mAb) therapeutics are now showing clinical and commercial success after intense research and development over the past 30 years [1]. To exploit the ability of antibodies to bind specifically to biologic targets, research and development have largely focused on developing mAbs against cell surface oncogenic receptor. To date, more than ten mAbs have been approved by the U.S. Food and Drug Administration to treat cancer, and more than 100 mAbs are still under investigation in clinical trials in patients with cancer [2, 3]. However, these anti-cancer antibodies do not necessarily cure cancer. Long-term treatment with mAbs can lead to intrinsic or acquired resistance [4]. Cell surface receptors such as human epidermal growth factor 1(HER1), human epidermal growth factor receptor 2 (HER2), and CD20 as well as growth factors such as vascular endothelial growth factor (VEGF) and interleukin-6 (IL-6) are used as tumor targets of mAbs; however, these targets are also found in normal cell proliferation and survival and thus are not ideal. Also, multiple clinical trials of therapies targeting vascular endothelial growth factor A or its receptors in patients with a variety of cancers have associated those therapies with only modest improvements in progression-free or overall survival [5].

The resistance of cancer stem cells to conventional mAb therapies, including mAbs targeting surface receptors, is a major obstacle to the successful treatment of malignant disease. There is an urgent need to understand the resistance of cancer to these therapies and to develop antibodies to overcome this resistance. As such, tumor-specific cell surface vimentin (CSV) was investigated in the present study as a therapeutic target. Vimentin is a multifunctional intermediate filament protein that interacts with several other proteins and participates in diverse cellular functions. Vimentin is expressed mainly in cells of mesenchymal origin and is often used as a marker for epithelial–mesenchymal transition (EMT) [6]. The overexpression of vimentin has been reported in various tumor cell lines, including breast cancer, central nervous system tumor, prostate cancer, malignant melanoma, and lung cancer cell lines [7]. Vimentin overexpression has also been associated with increased cancer cell growth, invasion, and migration, suggesting that vimentin participates in the promotion of these tumorigenic events and would serve as an effective target for cancer therapy. It has been reported that vimentin can also be expressed on the surfaces of various cancer cells [7–10] and can be secreted under certain conditions on endothelial cells [11, 12].

Our previous study of a novel linear vimentin-binding minipeptide and virus nanoparticles for CSV-targeted tumor-specific therapy indicated that vimentin is expressed on the surface of cancer stem cells (CSCs) and is internalized upon contact with specific ligands [7, 8]. Additionally, we have reported the discovery of CSV as a universal circulating tumor cell (CTC) marker by using a mAb, 84–1, that was generated to detect CSV on CTCs in patients with sarcoma or breast cancer [13–15]. However, it remains unknown whether CSV can serve as a therapeutic target in CSV-expressing tumor cells. Here, for the first time, we describe a novel mAb, 86C, which binds CSV on cancer tumor initiating and leads to the internalization of the vimentin and the consequent apoptosis of target cells. This CSV-targeting mAb was intensively investigated *in vitro* and *in vivo* as a treatment for glioblastoma multiforme (GBM).

## RESULTS

### CSV is expressed on GBM tumor initiating cells

**Figure 1:**
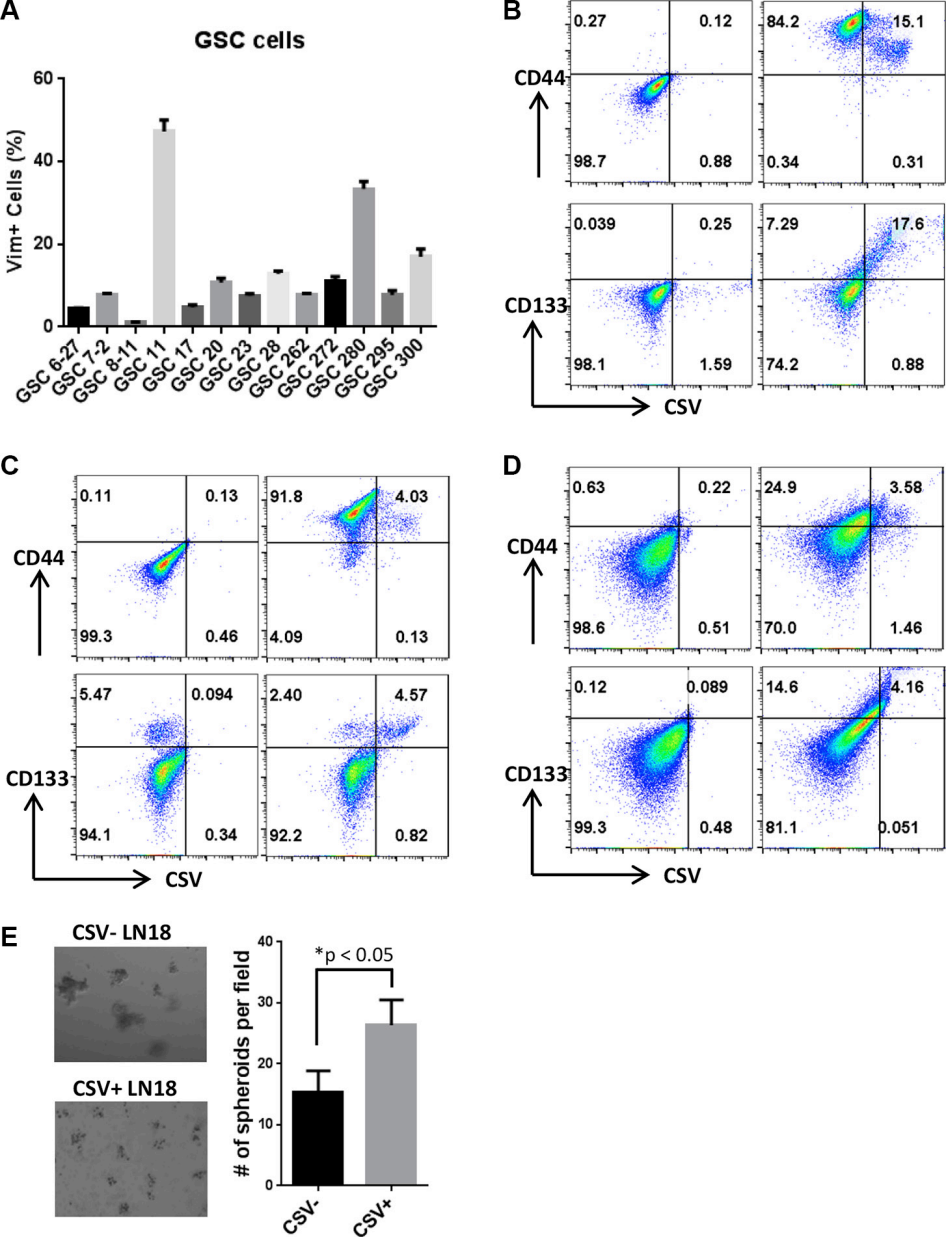
CSV is expressed primarily on GBM TICs (**A**) CSV expression on GSC cells. Cells were stained with 84-1 primary antibody and Alexa Fluor 405–conjugated secondary antibody or isotype control. CSV expression was determined by flow cytometry. Data are presented as mean ± standard error (*n* = 3). The co-expression of CSV and CSC markers CD133 and CD44 was found on human GBM LN18 cells (**B**) and GL261 cells (**C**) using flow cytometry. Results are representative of three independent experiments. (**D**) The co-expression of CSV and CSC markers CD133 and CD44 was found on the tumor cells of a GBM patient using flow cytometry. Results are representative of three independent experiments. (**E**) Images and numbers of spheres formed in Matrigel by the sorted CSV+ and CSV− LN18 cells on day 9.

Unlike intracellular vimentin, which is found in both cancer cells and normal mesenchymal cells, CSV is tumor specific. CSV has been found primarily on cancer cells such as CTCs and breast cancer cells [7, 8, 17], but CSV expression on tumor initiating cells (TICs) is not known. We screened a panel of well characterized GSC cells and found that CSV was universally expressed on all GSC cells tested, including GSC6-27, GSC7-2, GSC8-11, GSC11, GSC17, GSC20, GSC23, GSC28, GSC262, GSC272, GSC280, GSC295, and GSC300 (Figure 1A). On the basis of this result, we hypothesized that CSV-expressing GBM cells have the properties of TICs.

Current well-known cell surface markers of CSCs are developmental self-renewal pathway receptors and other receptors including CD44 and CD133 [18]. CD133 in particular is a marker for CSCs of several types of carcinomas such as sarcomas, melanoma, and highly aggressive brain tumors, including GBM [18]. To ascertain the association of CSV expression with CSC markers, the human GBM line LN18 cells were co-stained with the CSV-specific antibody 84–1 and the CSC markers CD133 and CD44; and CSV+CD133+ and CSV+CD44+ cells were analyzed using flow cytometry (Figure 1B). Most CSV-expressing cells showed CD133 expression (95% of those cells) or CD44 expression (98% of those cells), suggesting that CSV-expressing cells have TIC properties. The co-expression of CSV and the CSC markers CD133 and CD44 was also found on the mouse GBM cell line GL261 (Figure 1C). Importantly, tumor cells from a patient with GBM co-expressed CSV and CD133 (Figure 1D).

One biologic property of human TICs is the formation of cellular spheroids. To detect this property in CSV+ GBM cells, LN18 cells were flow sorted into CSV+ and CSV- cells using the CSV-specific mAb 84–1 and mouse immunoglobulin G (IgG) Microbeads. The sorted CSV+ and CSV- LN18 cells were then seeded onto Matrigel and monitored for spheroid formation for 9 days. The CSV+ LN18 cells formed significantly more spheroids (26.33 ± 2.404) relative to CSV- LN18 cells did (15.33 ± 2.028) (*P* = 0.0249, Figure 1E). However, the mean size of the spheroids formed by the CSV+ LN18 cells was smaller than the spheroids formed by the CSV- LN18 cells. This size difference was not abnormal; the binding of 84–1 to the CSV on tumor cells during CSV+ cell sorting lasts 2 days and thus may delay spheroid formation (Supplementary Figure S1). Taken together, these findings indicate that the expression of CSV on cancer cells is associated with TICs.

### Cell death due to the CSV-specific mAb 84-1 is cell line specific

Our laboratory has reported that CSV detected by 84–1 serves as a universal marker for CTCs from mesenchymal and epithelial tumors regardless of the tissue origin of the tumor [13–15]. Here, we tested the effect of treatment with 84–1 on direct tumor cell killing, using confluent monolayers of various tumor cell lines: human GBM cell lines (LN18, U251, and U87), mouse GBM cell lines (GL261 and DBT), and GSC cells (GSC11, GSC280, and GSC300). Significantly decreased viability was seen only in the LN18 cells after 84–1 treatment, but no significant effect on viability was seen after 84–1 treatment in the other cell lines (Supplementary Figure S2) indicating that the 84–1 antibody treatment had a tumor cell line–specific effect, although 84–1 detects CSV across different types of tumors. Therefore, additional mAbs against CSV were screened.

### CSV-specific mAb 86C targets tumor cells across GBM cell lines

**Figure 2:**
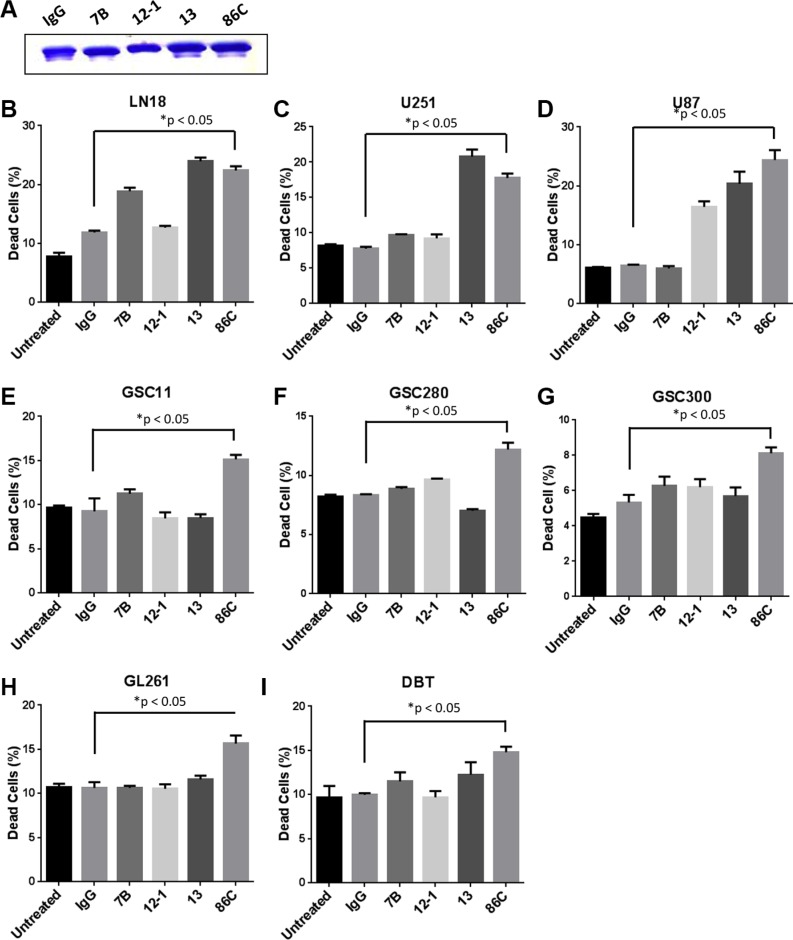
CSV-specific mAb 86C targets tumor cells across cell lines The antibodies IgG, 7B, 12, 13, and 86C (2 μg/mL for all, **A**) were used to treat human GBM cell lines (**B**–**D**), GSC cells (**E**–**G**), and mouse GBM cell lines (**H** and **I**), for 24 hours. Cells were collected and the dead cell population was analyzed using flow cytometry. Data are presented as mean ± standard error (*n* = 3). *P < 0.05 versus IgG treatment. Student *t* test.

Since the CSV-specific mAb 84–1 showed a limited role in direct tumor cell killing, we screened multiple other CSV-targeting mAbs from hybridoma fusion as described previously [13]. The highly specific CSV-targeting mAbs 7B, 12–1, 13, and 86C were selected for further direct cell death analysis. The human GBM cells LN18 were used for the initial screen which was incubated with low doses of these antibodies (2 μg/ml) (Figure 2A) and control mouse IgG (2 μg/ml), for 24 hours. The cell viability, as analyzed by flow cytometry, was significantly decreased by all antibodies except 12–1 (Figure 2B). Upon screening additional glioma cell lines and GSC cells, the mAB 86C, now designated as anti-CSV, demonstrated the most robust and universal GBM killing (Figure 2C–2I). Significantly decreased viability was seen across these different GBM cell lines after 86C treatment, while the rest of the antibodies showed cell line–specific effects. This result suggests that the mAb 86C can serve as a therapeutic antibody for GBM cell killing.

### 86C-mediated tumor cell death response is dose dependent and tumor cell specific

**Figure 3:**
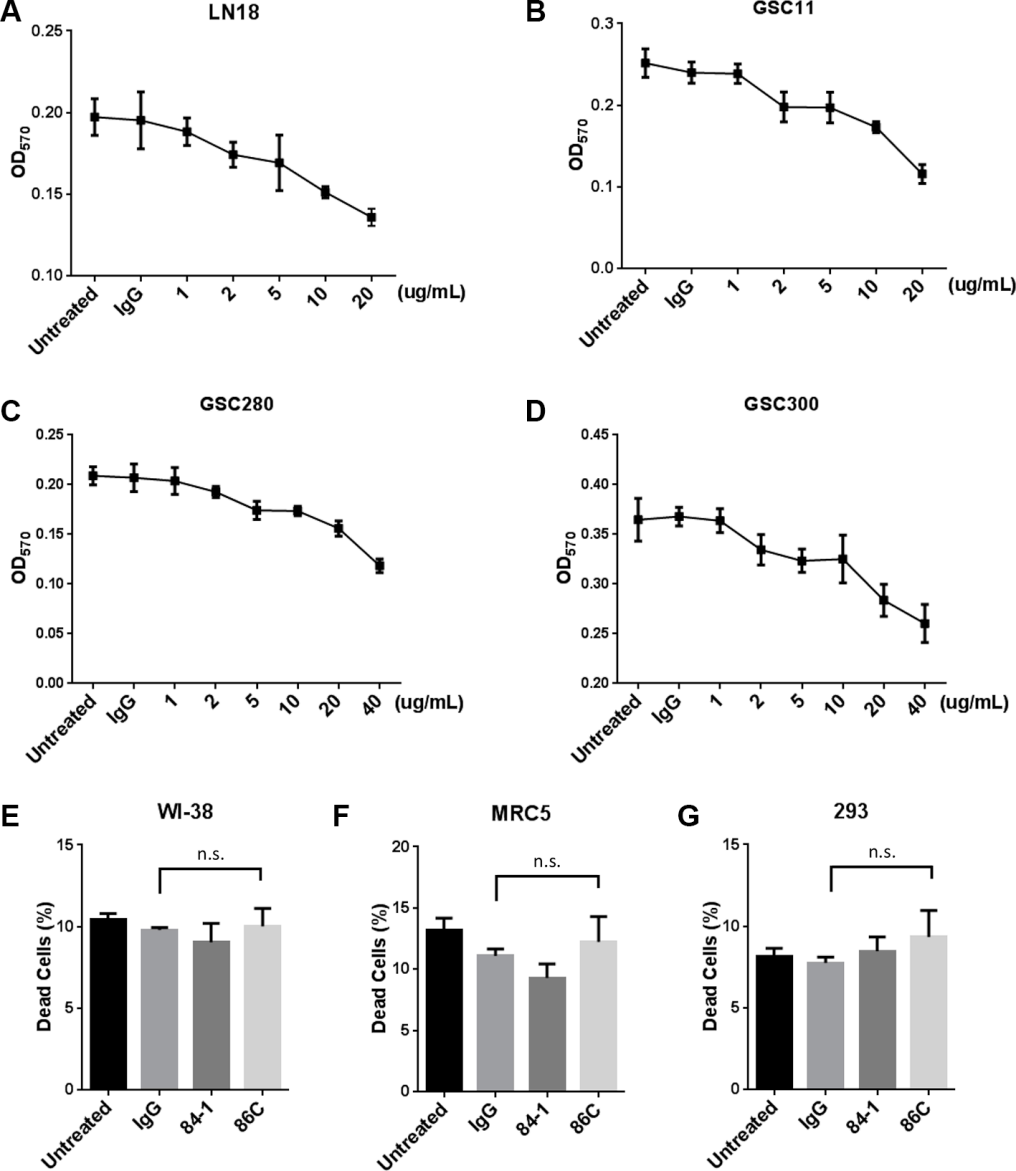
86C-mediated tumor cell death response is dose dependent and tumor cell specific Confluent LN18 (**A**), GSC11 (**B**), GSC280 (**C**), or GSC300 (**D**) cells were incubated with varying concentrations of 86C antibody (1 μg/ml to 20 μg/ml) and control IgG (20 μg/ml), and cell viability was assessed at 24 hours using an MTT assay. Data are presented as mean ± standard error (*n* = 3). Normal lung fibroblastoma cell lines (WI-38 (**E**) and MRC5 (**F**)) and human embryonic kidney 293 cells (**G**) were incubated with 2 μg/ml IgG, 84-1, or 86C for 24 hours. Cell viability was analyzed using an MTT assay. Data are presented as mean ± standard error (*n* = 3). n.s. versus IgG treatment. Student *t* test.

To investigate the efficiency and specificity of the 86C antibody in cell killing, confluent LN18 cells, GSC11 cells, GSC280 cells, and GSC300 cells were incubated with varying concentrations of 86C (1 μg/ml to 20 μg/ml) and cell viability was assessed after 24 hours using an 3-(4,5-Dimethylthiazol-2-yl)-2,5-Diphenyltetrazolium Bromide (MTT) assay. Higher concentrations of 86C reduced cell survival; fewer than 20% of cells were viable after treatment with 20 μg/ml 86C for 24 hours (Figure 3A–3D). To determine whether 86C-mediated cell death is cancer cell specific, we also tested normal cells from the human lung fibroblast cell lines WI-38 and MRC5, and the human embryonic kidney 293 cell line and found no killing (Figure 3E–3G), suggesting that the 86C-mediated cell death is specific to cancer cells.

### Characterization of the 86C mAb

**Figure 4:**
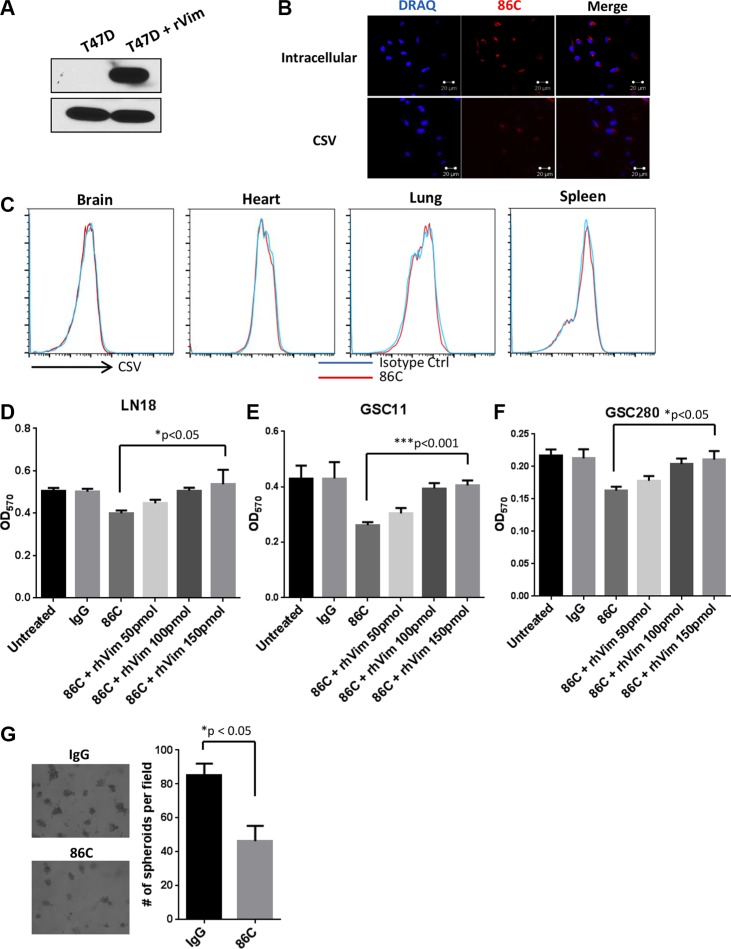
Characterization of the 86C mAb (**A**) T47D cell lysates and T47D cell lysates with 50 ng of rhVim protein were subjected to immunoblotting to detect vimentin using the 86C antibody. GAPDH was used as a loading control. (**B**) Immunostaining of CSV or cytosolic vimentin (red) expression in LN18 cells. Nuclei (blue) were stained with DRAQ5. (**C**) Normal mouse cells dissociated from brain, heart, lung, and spleen tissues were stained with mouse IgG or 86C and Alexa Fluor 405–conjugated secondary mouse-IgG. 86C staining was analyzed using flow cytometry. Results are representative of three independent experiments. LN18 (**D**), GSC11 (**E**), or GSC280 (**F**) cells were incubated with various concentrations of rhVim for 1 hour before treatment with 86C to neutralize the impact of 86C on the cells. Cells were collected, and the dead cell population was analyzed using flow cytometry. Data are presented as mean ± standard error (*n* = 3). *p = 0.02 versus 86C+rhvim 150pmol treatment. Student t test. (**G**) LN18 cells were suspended on Matrigel in the presence of 86C (10 μg/ml) or IgG. The spheres formed in Matrigel were imaged and counted on day 7.

The vimentin-specific mAb 86C was generated following immunization with a recombinant vimentin protein fragment. To confirm that this antibody can detect vimentin expressed both intracellularly and on the cell surface, 86C was used in a Western blot analysis using total protein extracts from the vimentin-null breast cancer cell line T47D. This cell line served as a perfect negative control to determine 86C specificity owing to its lack of any endogenous vimentin expression. T47D cell lysates spiked with 50 ng of recombinant human vimentin protein (rhVim) served as a positive sample for determining 86C specificity. A strong immunoreactive band at the predicted molecular weight of vimentin (53 kDa) was detected with 86C in the lane containing rhVim but not in the lane containing T47D cell lysate only (Figure 4A). Moreover, intracellular and cell surface vimentin was detected in LN18 cells using immunofluorescence, indicating that 86C recognizes vimentin protein (Figure 4B). Taken together, these results show that 86C recognizes both CSV and intracellular vimentin. More importantly, 86C showed specific binding to cancer cells but not to any normal mouse cells disassociated from various normal tissues, including brain, lung, liver, and spleen tissues (Figure 4C).

### CSV is responsible for 86C-induced cell death in GBM cells

To determine the role of CSV during 86C-induced cancer cell death, LN18, GSC11 and GSC280 cells were incubated with various concentrations of rhVim for 1 hour before treatment with the 86C mAb to neutralize the impact of 86C to the cells. Blocking the 86C with rhVim led to a dose-dependent increase in cell viability (Figure 4D–4F). This result shows that the CSV on tumor cells plays an important role in 86C-mediated tumor cell death. To validate this observation, the role of CSV during spheroid formation in LN18 cells on Matrigel was tested. LN18 GBM cells were plated on Matrigel and cultured for 7 days in IgG- or 86C-conditioned media. Compared with the IgG treatment, the 86C treatment significantly inhibited the number of spheroids formed from seeded LN18 cells (Figure 4G). Spheroids also were smaller in the 86C-treated samples than in the IgG-treated samples. To further confirm the importance of CSV in 86C-mediated tumor cell death, > 90% intracellular vimentin was knocked down by the stable expression of vimentin shRNAs in LN18 cells (Supplementary Figure S3A), which did not affect CSV expression on the cell surface (Supplementary Figure S3B). Indeed, the vimentin-knockdown LN18 cells still showed reduced cell viability after 86C treatment owing to their CSV expression (Supplementary Figure S3C). These results suggest that CSV is responsible for the 86C-induced cell death response in LN18 cells.

### Rapid internalization of 86C is associated with induction of cell apoptosis and inhibition of cell proliferation

**Figure 5:**
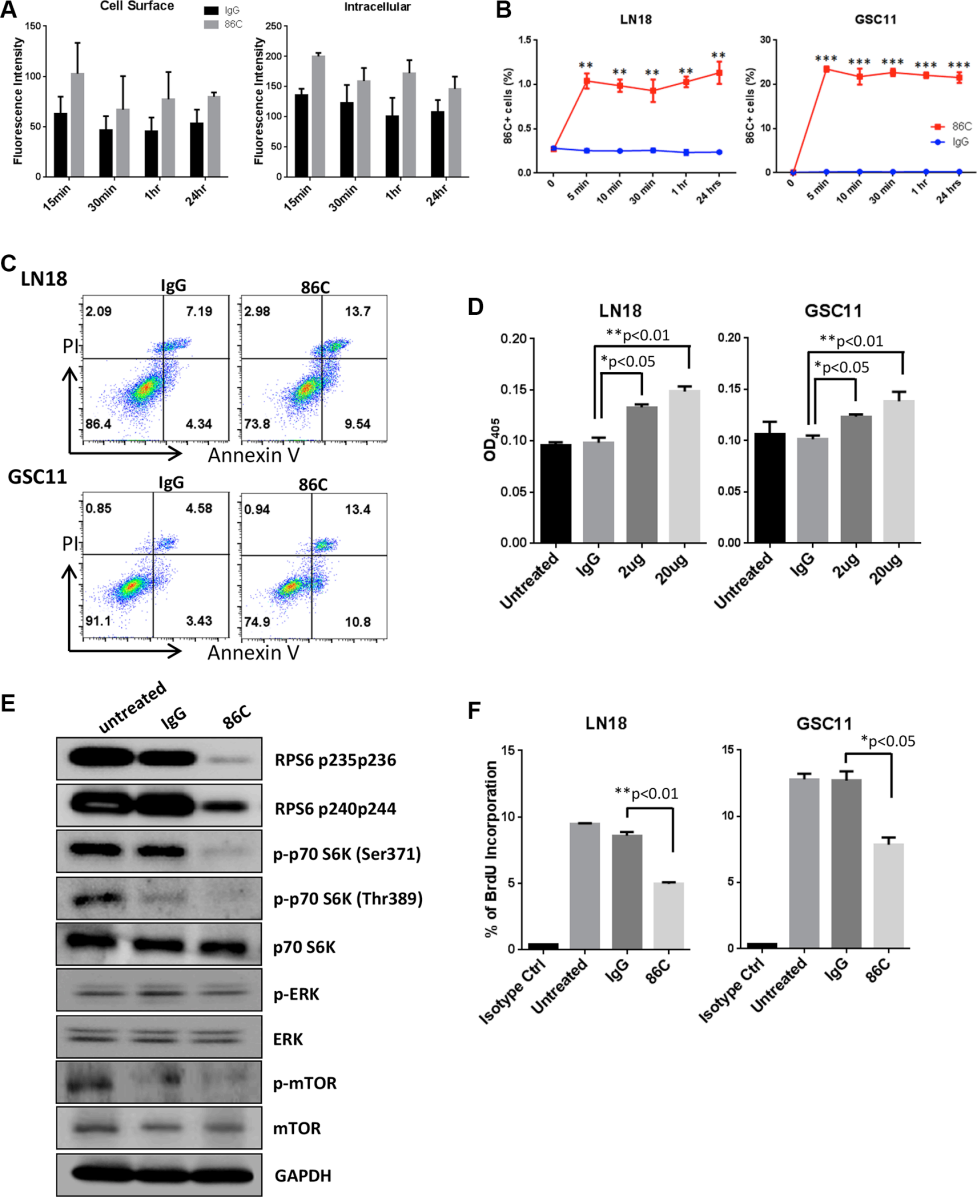
Rapid internalization of 86C is associated with induction of cell apoptosis and inhibition of cell proliferation (**A**) LN18 cells were incubated with 86C for 15 minutes, 30 minutes, 1 hour, or 24 hours and were fixed and permeabilized for immunofluorescent detection. The 86C in the cells was stained with Alexa Fluor 647–conjugated IgG, and total fluorescence intensity was measured at 650 nm excitation and 665 nm emission. 86C on the LN18 cell surface was also stained with Alexa Fluor 647–conjugated IgG without permeabilization. (**B**) Cells cultured with 86C for 5 minutes, 10 minutes, 30 minutes, 1 hour, or 24 hours were fixed using 2% paraformaldehyde. After fixation, cells permeabilized were stained with Alexa Fluor 405–conjugated anti-mouse IgG to trace 86C in the cells and analyzed using flow cytometry. Data are presented as mean ± standard error (*n* = 3). **P < 0.01 or ***p < 0.005 versus IgG treatment. Student *t* test. (**C**) LN18 and GSC11 cells were treated with 2 μg/ml IgG or 86C for 24 hours. Then, single-cell suspensions were stained with Annexin V and propidium iodide to determine proportions of necrotic and apoptotic cells by flow cytometry. (**D**) Caspase-3 assay of LN18 and GSC11 cells treated with different concentrations of 86C. The reduction in cell viability correlated with an increase in caspase-3 activity, which was statistically significant at 2 μg/ml (*P* < 0.05). (**E**) LN18 and GSC11 cells were treated with 10 μg/ml 86C for 24 hours. Cells were subjected to lysis, and cell lysates were subjected to immunoblotting to detect phosphor-RPS6, phosphor-p70S6K, p70S6K, phosphor-ERK, ERK, phosphor-mTOR, and mTOR. GAPDH was used as a loading control. (**F**) BrdU at 10 μM was incorporated into LN18 and GSC11 cells treated with 10 μg/ml 86C for 2 hours. Incorporated BrdU was stained and analyzed using flow cytometry. Data are presented as mean ± standard error (*n* = 3). *P < 0.05 versus IgG treatment. Student *t* test.

Antibody binding to a cell surface antigen may stabilize the antigen-antibody complex outside the cell membrane or modulate the complex, which is dependent on cellular metabolism and thereby manifest itself as internalization or shedding [19]. To evaluate the internalization of the 86C mAb, LN18 cells were incubated with 86C for 15 minutes, 30 minutes, 1 hour, or 24 hours, fixed, and permeabilized for immunofluorescence detection. The 86C in the cells was stained with Alexa Fluor 647–conjugated IgG, and total fluorescence intensity was measured at 650 nm excitation and 665 nm emission. 86C on the LN18 cell surface was also stained with Alexa Fluor 647–conjugated IgG without permeabilization. Cell surface binding and internalization of 86C showed the highest intensities at 15 minutes after 86C treatment in LN18 cells (Figure 5A). To further demonstrate the internalization of 86C during incubation, LN18 and GSC11 cells were incubated with 86C for various times. After then, intracellular 86C in the cells was stained with an Alexa Fluor 405–IgG antibody and analyzed by flow cytometry. As shown in Figure 5B, intracellular 86C was detected in LN18 and GSC11 cells during incubation. After 5 minutes of incubation, intracellular 86C in LN18 and GSC11cells showed similar staining with other time points, suggesting the rapid internalization of 86C. Taken together, these data indicate that 86C binds to vimentin on the GBM tumor cell surface and rapidly internalizes upon interaction with CSV with time-dependent kinetics.

The internalization of vimentin may induce cell responses such as cell apoptosis. To determine whether the induction of apoptosis accounts for the reduction in cell viability after 86C treatment, we performed propidium iodide/Annexin V double staining. Results indicated significant apoptosis after treatment of LN18 cells and GSC11 cells with 10 μg/ml 86C (Figure 5C). To confirm apoptosis as the general mechanism of cell death, we used a caspase-3 activity assay as an indicator of apoptosis. A significant increase in caspase-3 activity suggested that the mechanism of cell death was indeed induction of apoptosis (Figure 5D).

To investigate changes in protein expression after 86C treatment, a reverse phase protein array (RPPA) analysis on LN18 samples treated with 86C using 305 different antibody probes was performed. We found significant protein expression changes in 86C-treated LN18 cells. Specifically, the phosphor–ribosomal protein S6 (RPS6) protein was dramatically reduced by 86C treatment in LN18 cells (data not shown). To help determine the specific molecular mechanisms that are altered in 86C-treated LN18 cells, we interrogated the total proteins for which the probes showed significant reductions after 86C treatment; we observed a reduction of signaling events regulating the RPS6 pathway, which is significantly involved in protein synthesis in eukaryotic cells (Figure 5E). Reduction of this pathway biologically corresponded with the inhibition of RPS6 phosphorylation at serines 235, 236, 240, and 244 and of p70S6K phosphorylation at serine 371 and threonine 389. The reduction of these phosphorylated proteins highlights the possibility of inhibition of cell proliferation and induction of apoptosis as therapeutic mechanisms of 86C treatment in LN18 glioma cells. The inhibition of LN18 and GSC11 cell proliferation by 86C treatment was confirmed using a bromodeoxyuridine (BrdU) incorporation assay (Figure 5F). A possible model for this molecular mechanism is depicted in Figure 7 and illustrates that the suppression of RPS6 phosphorylation via the 86C antibody against CSV on tumor cells may inhibit cell proliferation and induce cell apoptosis.

### 86C reduces tumor size in mice

**Figure 6:**
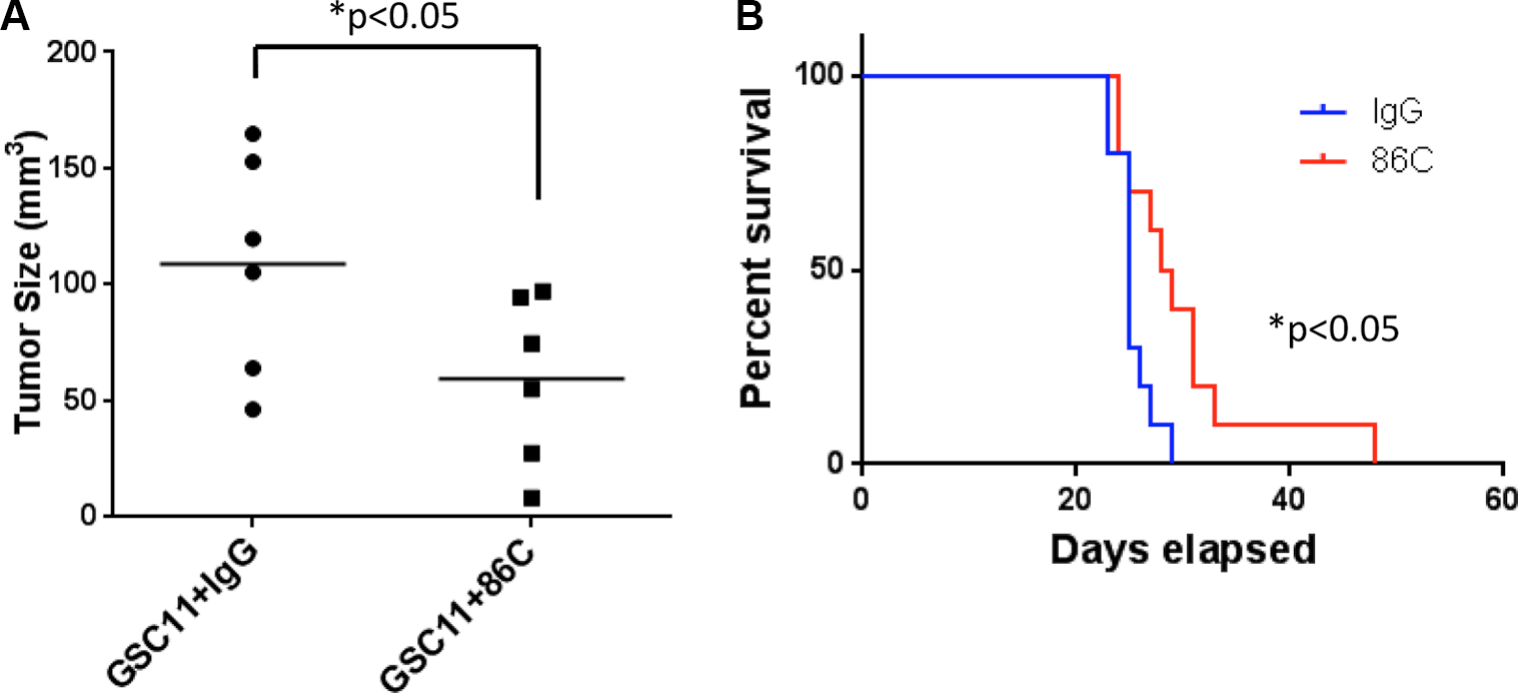
86C reduces tumor size in mice (**A**) Volumes of GSC11 tumors in NSG mice. GSC11 cells with IgG or with 86C were injected into both side flanks of NSG mice (*n* = 6). The volumes of subcutaneous tumors were measured on day 15 of injection. Two-way analysis of variance was used to calculate the two-sided *P value*s. Error bars are standard deviations. (**B**) The survival of GSC11glioma-bearing mice (*n* = 10). The GSC11 cells with IgG or 86C were intracerebrally injected in NSG mice and the survival of mice was monitored. Log-rank test was used to compare overall survival between groups.

To examine the 86C antibody therapeutic approach in a more relevant model for future clinical applications, we tested the treatment in mice. To assess whether the 86C antibody exerts a therapeutic effect, immune-deficient NOD/SCID gamma (NSG) mice were subcutaneously injected with GSC11 tumor cells with IgG or 86C. The progression of tumor burden was evaluated as directed measurements of tumor size. Compared with the mice injected with GSC11 cells with control IgG, the mice injected with cells with 86C showed a significantly lower rate of tumor growth (Figure 6A). These data indicated that 86C antibody treatment inhibits tumor growth in GSC11 tumor–bearing mice. Furthermore, immune-deficient NOD/SCID gamma (NSG) mice harboring intracerebral GSC11 tumor cells with IgG or 86C were monitored for survival. The median survival time was statistically significantly longer in mice injected with GSC11 cells with 86C relative to those injected with GSC11 cells with IgG control (29 days and 25 days, respectively, *p* = 0.02) (Figure 6B). Collectively, these results show that the 86C antibody has the potential to effectively treat GBM.

**Figure 7:**
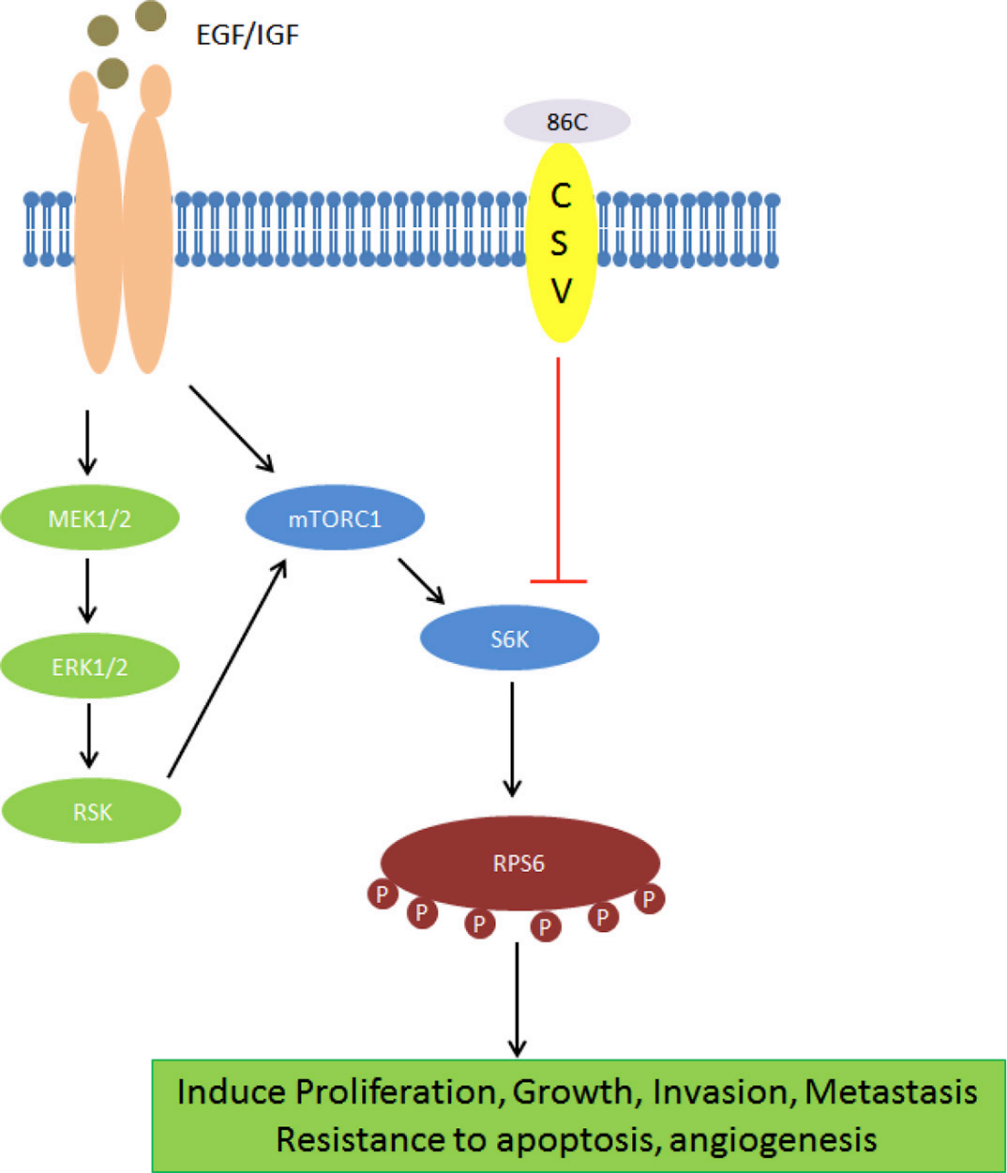
A possible model for 86C mechanism The suppression of RPS6 phosphorylation *via* the 86C antibody against CSV on tumor cells may inhibit cell proliferation and induce cell apoptosis.

## DISCUSSION

mAb therapies are proving useful in the treatment of diseases such as cancer, but the success of these therapies depends on the identification of suitable target proteins that play important roles in cancer progression and are homogenously over-expressed and accessible on the surface of tumor cells [20].

We showed that targeting CSV with the 86C antibody specifically and significantly induces GBM cell apoptosis *in vitro* and arrests GBM progression *in vivo*. In addition, we found that CSV-expressing GBM cells have properties of TICs, including the propensity to form stable colonies. Additionally, we show the co-expression of CSV and CD133. TICs appear to be ideal targets, particularly for postsurgical treatments, because these cells preferentially locate to the invasive front of highly aggressive tumors, thereby often escaping surgical resection [21, 22]. Our results show that CSV is found on GBM TICs, suggesting the potential of CSV as a target for treating aggressive cancer cells.

In addition, the expression of CSV in cell lines derived from various types of cancers, including breast cancer, colon cancer, neuroblastoma, osteosarcoma, and glioma, suggests that an anti-CSV antibody could be used to treat various cancer types. Indeed, our study showed an effect of 86C against CSV-expressing cancer cells *in vitro* as well as *in vivo* in subcutaneous and intracranial models of brain tumors.

A potential mechanism of treatment failure with an anti-CSV therapeutic approach is persistence of CSV expression. Specifically, we found CSV expression levels were ~10% on anti-CSV-treated LN18 cancer cells (Supplementary Figure S4). This correlated with the associated cell viability being decreased by ~80% with 86C treatment compared with control IgG. This decrease in viability correlated with an increase in caspase-3 activity, indicating activation of apoptosis, and with a decrease in spheroid formation on Matrigel assays, providing an *in vitro* indication of a blocking effect of 86C on TICs. The therapeutic index of the anti-CSV antibody certainly could be enhanced especially since the *in vitro* MTT assays indicated that 86C induced selective killing of cancer cells but not normal cells. The mechanisms responsible for this cell killing by 86C are currently not well characterized, but several possibilities could be envisioned from this study. A small fraction of intracellular vimentin may be released, externalized, and displayed on the surfaces of cancer cells by unconventional secretion. The binding of 86C to CSV may then trigger rapid internalization of an 86C-vimentin complex by the cancer cells. Once inside the cell, the 86C-vimentin complex is capable of activation of apoptosis signaling, leading to apoptosis and inhibited cell proliferation. This approach clearly contrasts with approaches that target receptor tyrosine kinases such as epidermal growth factor receptor (EGFR) and human epidermal growth factor receptor 2 (HER2), in which preventing receptors from interacting with ligands (soluble growth factors) and inhibiting tyrosine kinase activity are proving successful [23, 24].

In conclusion, CSV is a promising therapeutic target in the treatment of GBM and the development of anti-cancer therapies. The CSV-specific 86C mAb therapy causes internalization of CSV and induces further antibody-dependent signaling that leads to tumor cell apoptosis and inhibition of tumor cell proliferation; thus, 86C is a promising targeted treatment for CSV-expressing tumors. Additionally, 86C may be beneficial in the treatment of highly aggressive CSV+ tumors with TICs features, particularly if administered into surgically created resection cavities of brain tumors. The efficacy of 86C may be enhanced by simultaneous targeting of TICs using other surface markers or by combining this targeted therapy with other approaches. Furthermore, 86C combination treatment with the current standard care including radiotherapy and chemotherapy for GBM patients may enhance the therapeutic efficacy of treatment. Finally, the effects on tumor-killing in the presence of 86C should be further confirmed using humanized NSG mice to prove that immune system of host does not affect the anti-tumor effect of 86C, which is useful information for human patients and the future of preclinical therapeutic development. Also further studies are needed for 86C to attain clinical applicability after humanization of the antibody.

## MATERIALS AND METHODS

### Ethics statement

The mice used in this study were maintained under the National Institutes of Health guidelines and euthanized according to procedures approved by the Institutional Animal Care and Use Committee of The University of Texas MD Anderson Cancer Center.

### Cell lines and cell culture

GBM stem cell lines were provided by Dr. Frederick Lang at The University of Texas MD Anderson Cancer Center (2014) (Houston, TX) and cultured in serum-free Dulbecco's modified Eagle medium (DMEM) supplemented with epidermal growth factor (EGF, 20 ng/ml), basic fibroblast growth factor (bFGF, 20 ng/ml), and 2% B27 (Life Technologies, Grand Island, NY). The DBT cell line was kindly provided by Dr. Leonid Metelitsa (2012) (Baylor College of Medicine, Houston, TX). The glioma cell lines LN18, U87, U251, and GL261 and the fibroblast cell lines MRC5 and WI-38 were obtained from the ATCC (Rockville, MD) within the last 5 years. The glioma cells and fibroblast cells were cultured in DMEM/F12 (Sigma-Aldrich, St. Louis, MO) supplemented with 10% fetal bovine serum and 10 U/ml penicillin and streptomycin (Life Technologies) at 37°C in 5% CO_2_. Cells were detached using 1 mM ethylenediaminetetraacetic acid (EDTA) in phosphate-buffered saline solution (PBS) and used for further experiments. No further authentication was conducted in our laboratory for cell lines.

### Flow cytometry

The single-cell suspension was blocked for 10 minutes at room temperature with FcR blocker (Miltenyi Biotec Inc.) in a 1:1000 dilution and then incubated with 0.4 μg of 84–1 for 15 minutes at room temperature. After being washed, cells were incubated with 0.5 μg of goat anti-mouse Alexa Fluor 405–conjugated secondary antibody in PBS plus 2% serum for 15 minutes in the dark at room temperature. Cells were analyzed on an Attune flow cytometer (Life Technologies), and the results were evaluated using FlowJo 10.0 software (Tree Star, Inc., Ashland, OR).

Heart, brain, and lung tissue from a C57BL/6 mouse was dissociated with scissors and digested with liberase for 1 hour at 37°C. The digested preparation was filtered with a 40-μm nylon strainer. Spleen tissue from a mouse was homogenized gently in a 40-μm nylon strainer. Red blood cells were subjected to lysis with Gentra Puregene red blood cell lysis solution (QIAGEN, Hilden, Germany). Heart, brain, lung, and spleen cells (50,000 cells/sample) were stained with 86C primary antibody followed by goat anti-mouse Alexa Fluor 405–conjugated secondary antibody staining.

### Spheroid formation analysis

The spheroid formation of cells on Matrigel was analyzed. Briefly, 100 μl of 10 mg/ml Matrigel (BD Biosciences, Bedford, MA) was polymerized in each well of a chamber slide for 1 hour at 37°C. The Matrigel layer was then dehydrated overnight and re-hydrated 30 minutes before use with 100 μl of DMEM culture medium. Excess medium was carefully removed before the addition of 10^4^ CSV+ or CSV- LN18 cells. Cells were incubated on Matrigel for 9 days under serum-free DMEM supplemented with epidermal growth factor (EGF) (20 ng/ml) and basic fibroblast growth factor (bFGF) (20 ng/ml). The medium was changed every 2 days. Spheroid formation was assessed using a Nikon Eclipse E800 light microscope (Tokyo, Japan). To test the role of CSV during the formation of spheroids of LN18 on Matrigel, 10^4^ LN18 cells with either the 86C mAb (10 μg/ml) or IgG as a control were incubated on Matrigel for 7 days.

### *In vitro* cell viability assays

Cell viability assays were carried out to evaluate the therapeutic efficacy of 86C. The cancer cells were treated with IgG or 2 μg/ml 7B, 12–1, 13, 84–1, or 86C for 24 hours and then were harvested and stained with LIVE/DEAD Aqua Dead Cell Stain Kit (Life Technologies) according to the manufacturer's protocol. Stained cells were analyzed on an Attune flow cytometer (Life Technologies), and the results were evaluated using FlowJo 10.0 software.

### *In vitro* internalization assays using flow cytometry

Cells were cultured with IgG, or 86C for 5 minutes, 10 minutes, 30 minutes, 1 hour, or 24 hours and then fixed using 2% paraformaldehyde (Thermo Fisher Scientific). After fixation, cells permeabilized were stained with Alexa Fluor 405–conjugated anti-mouse IgG to trace intracellular 86C in the cells. Cells were analyzed on an Attune flow cytometer (Life Technologies), and the results were evaluated using FlowJo 10.0 software (Tree Star, Inc., Ashland, OR).

### Annexin V and propidium iodide staining

To determine apoptosis of cancer cells due to antibody treatment, LN18 and GSC11 cells were treated with 2 μg/ml IgG or 86C for 24 hours. After 24 hours of treatment, single-cell suspensions were prepared with cold PBS buffer. After two washes, cells (1 × 10^6^ cells/ml) were resuspended in 500 μl of Annexin V binding buffer (BioLegend, San Diego, CA). Aliquots (100 μl) of the cell suspension were incubated with 5 μl of Pacific blue–conjugated Annexin V (BioLegend) and 5 μl of propidium iodide solution (Biotium, Hayward, CA) for 15 minutes at room temperature in darkness. After staining, 400 μl of Annexin V–binding buffer was added to the cells, which were then immediately analyzed by flow cytometry.

### Caspase-3 activity

Caspase-3 activity, an indicator of apoptosis, was determined using a Caspase-3 Colorimetric Assay Kit (R&D Systems, Minneapolis, MN) according to the manufacturer's protocol. Cells were lysed using lysis buffer. Fifty microliters of the cell lysis was incubated with 5 μl of caspase-3 colorimetric substrate (DEVD-pNA) in Reaction Buffer with dithiothreitol (DTT) at 37^°^C for 1 hour. The caspase-3 activity was measured using a wavelength of 405 nm.

### Western blot analysis

To analyze protein expression, cells were lysed in radioimmunoprecipitation assay (RPPA) buffer (50 mM Tris-HCl, pH 7.4; 1% NP-40; 0.25% sodium deoxycholate; 150 mM NaCl; 1 mM EDTA) supplemented with a complete protease inhibitor cocktail (Roche, Basel, Switzerland). The same amount of proteins was separated by 12% sodium dodecyl sulfate polyacrylamide gel electrophoresis (SDS-PAGE) and transferred to nitrocellulose membranes using the iBlot gel transfer device (Thermo Fisher Scientific). The membranes were blotted with appropriate primary antibodies and horseradish peroxidase (HRP)–conjugated secondary antibody to detect the proteins of interest. The monoclonal anti-vimentin antibody 84–1 was produced in a mouse. The anti-RPS6-p235p236, anti-RPS6-p240p245, anti-p70S6K-p371, anti-p70S6K-p389, anti-p70S6K, anti-pERK, anti-ERK, anti-p-mTOR, anti-mTOR, and anti–glyceraldehyde 3-phosphate dehydrogenase (GAPDH) primary antibodies were from Cell Signaling Technology (Danvers, MA). The anti-mouse and anti-rabbit horseradish peroxidase (HRP)–conjugated secondary antibodies were from Santa Cruz Biotechnology (Dallas, TX).

### Tumor models

Logarithmically growing GSC11 cells were inoculated into the flanks of 6-week-old NSG mice (Stock No. 005557, The Jackson Laboratory) with 1 × 10^6^ cells suspended in 30 μl of IgG or 86C antibody containing PBS in the subcutaneous glioblastoma model. Tumors were measured on day 15 after inoculation, and tumor volumes were calculated using the formula π(length × width^2^)/8. The length represents the longest axis, and the width is perpendicular to the length. In the orthotropic glioblastoma model, logarithmically growing GSC11 cells were collected and washed with PBS. GSC11 cells were pre-incubated with 20 ug of IgG or 86C, mixed with equal volume of 3% methylcellulose in PBS. GSC11 cells (5 × 10^5^ and 20 ug IgG or 86C) in a total volume of 5 μl were injected intracerebrally into 6-week-old NSG mice (Stock No. 005557, The Jackson Laboratory) as previously described [16]. The mice were observed three times per week and monitored the survival.

### Statistical analysis

Results are expressed as mean ± standard deviation. Data were analyzed with GraphPad software (GraphPad Software, Inc., La Jolla, CA) using an unpaired two-tailed Student *t-test* to determine the significance of differences between groups. *P* < 0.05 was considered statistically significant.

## SUPPLEMENTARY MATERIALS FIGURES


