# Supplementary material for: Discovery of cell surface vimentin targeting mAb for direct disruption of GBM tumor initiating cells
Source: Oncotarget. 2016 Oct 4;7(44):72021–32. doi: 10.18632/oncotarget.12458 (PMC5342141; doi:10.18632/oncotarget.12458)
Supplement: Supplementary file 1 [file oncotarget-07-72021-s001.pdf]

Discovery of cell surface vimentin targeting mAb for direct disruption of GBM tumor initiating cells

Supplementary Materials

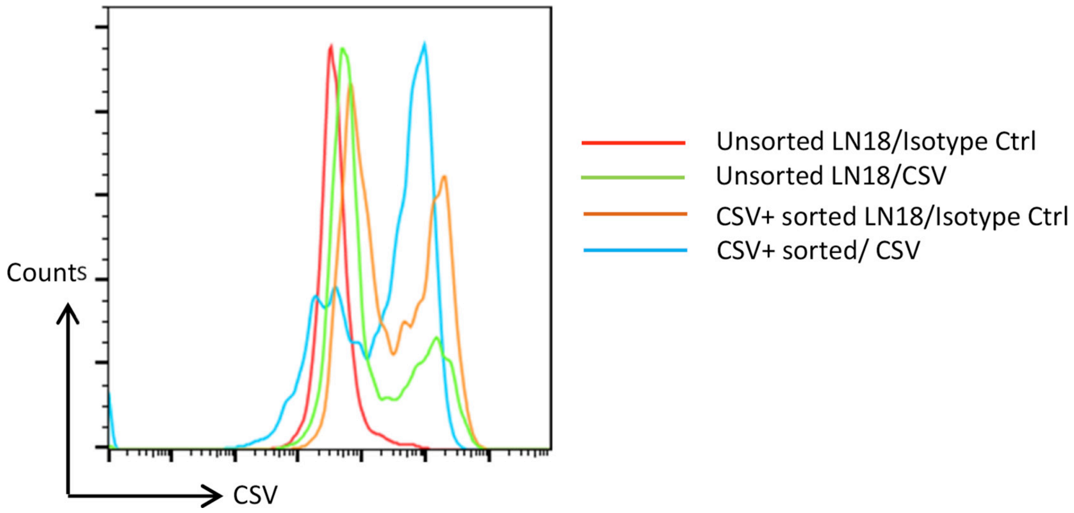

Supplementary Figure S1: The binding of 84–1 to the CSV on tumor cells during CSV+ cell sorting lasts 2 days. The CSV+ LN18 cells were incubated for 48 hrs. 48 hrs later, CSV+ LN18 cells and unsroted LN18 cells were stained with 84–1 primary antibody and goat anti-mouse Alexa Fluor 405–conjugated secondary antibody.

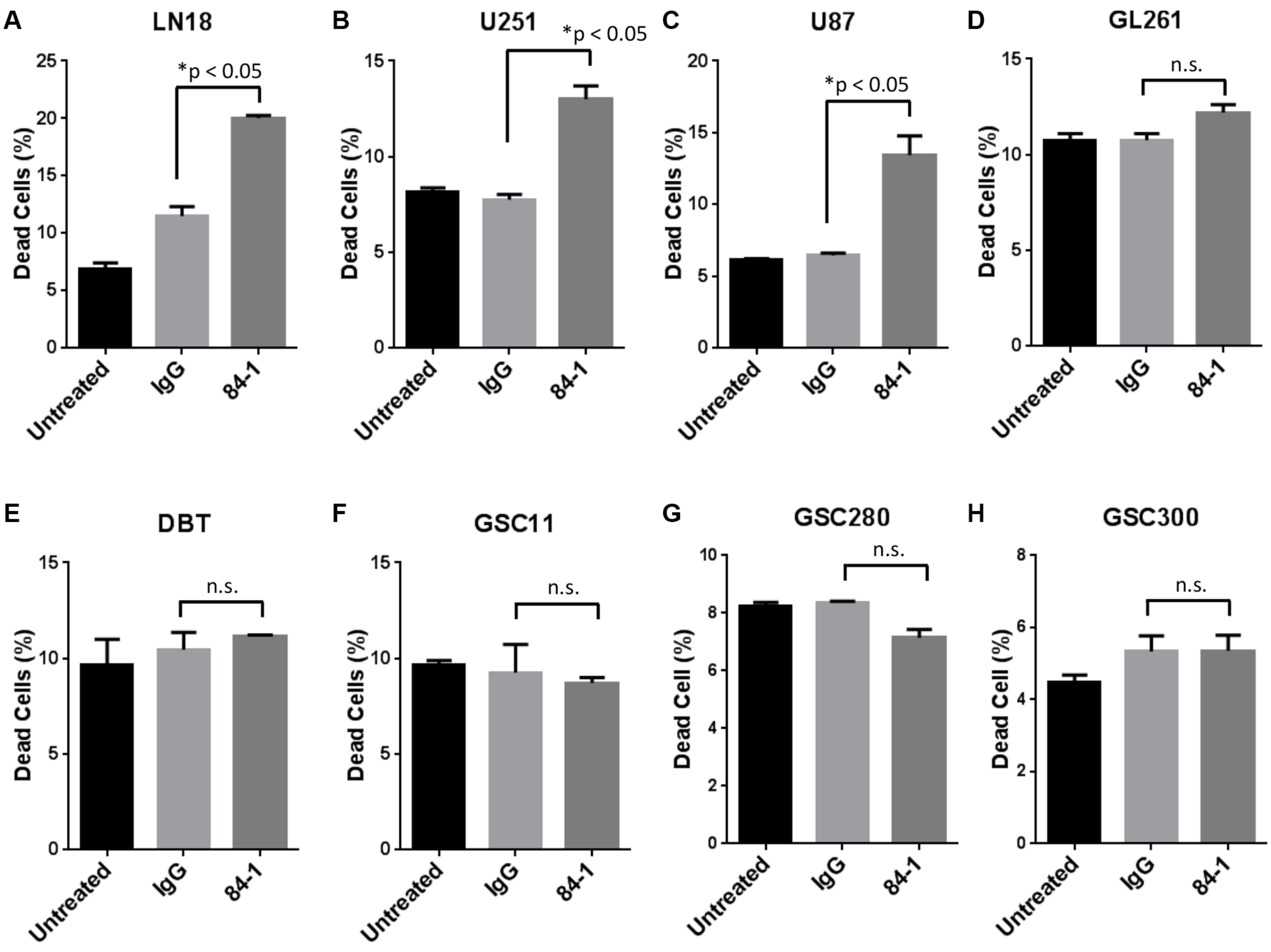

Supplementary Figure S2: Cell death due to the CSV-specific mAb 84-1 is cell line specific. The antibodies of 2 μg/mL IgG and 84–1 were used to treat human GBM cell lines (**A**–**C**), mouse GBM cell lines (**D** and **E**) and GSC cells (**F**–**H**), for 24 hours. Cells were collected and the dead cell population was analyzed using flow cytometry. Data are presented as mean ± standard error (n = 3). *$P < 0.05$ versus IgG treatment. Student t test.

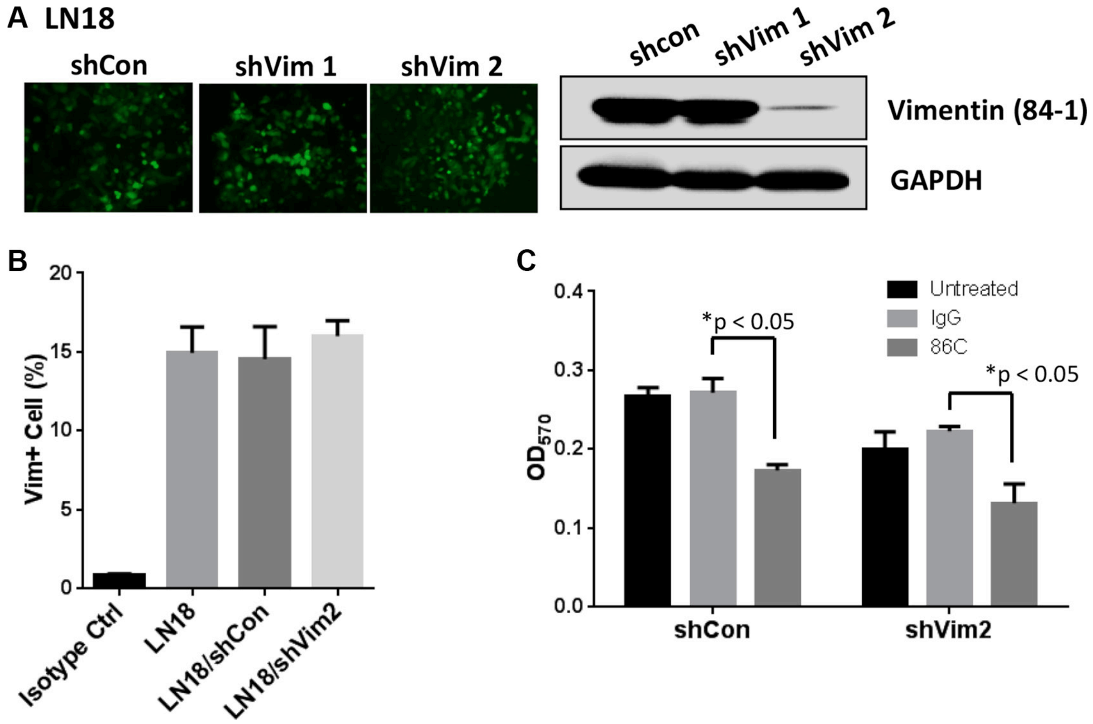

Supplementary Figure S3: Vimentin knockdown was not affect in 86C-mediated tumor cell death. (**A**) vimentin was knocked down by the stable expression of vimentin shRNAs in LN18 cells and GFP and vimentin expression were determined using microscope and western blotting. GAPDH was used as control. (**B**) CSV was stained using 84–1 and analyzed using flow cytometry. (**C**) Confluent LN18/shCon or LN18/shVim2 cells were incubated with 2 µg/ml 86C antibody, and cell viability was assessed at 24 hours using an MTT assay. Data are presented as mean ± standard error ($n = 3$). *$P < 0.05$ versus IgG treatment. Student t test.

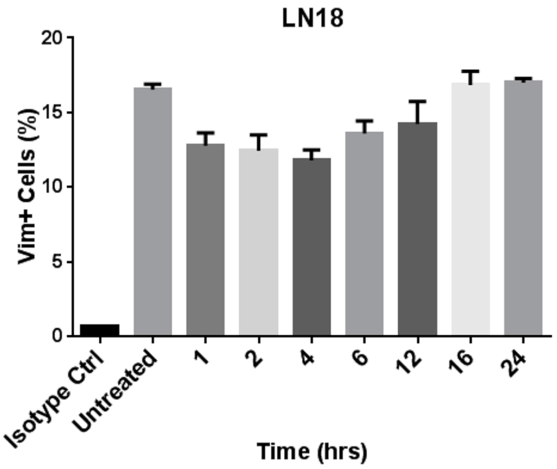

Supplementary Figure S4: CSV expression levels were ~10% on anti-CSV-treated LN18 cancer cells. Confluent LN18 cells were incubated with 2 µg/ml 86C antibody for variant time points and CSV expression was analyzed using flow cytometry. Data are presented as mean ± standard error ($n = 3$).
